# Towards Distributed Recycling with Additive Manufacturing of PET Flake Feedstocks

**DOI:** 10.3390/ma13194273

**Published:** 2020-09-25

**Authors:** Helen A. Little, Nagendra G. Tanikella, Matthew J. Reich, Matthew J. Fiedler, Samantha L. Snabes, Joshua M. Pearce

**Affiliations:** 1 re:3D Inc., 1100 Hercules STE 220, Houston, TX 77058, USA; helen@re3d.org (H.A.L.); matthew@re3d.org (M.J.F.); samantha@re3d.org (S.L.S.); 2 Department of Material Science and Engineering, Michigan Technological University, Houghton, MI 49931, USA; ngtanike@mtu.edu (N.G.T.); mjreich@mtu.edu (M.J.R.); 3 Department of Electrical and Computer Engineering, Michigan Technological University, Houghton, MI 49931, USA; 4 Department of Electronics and Nanoengineering, School of Electrical Engineering, Aalto University, 00076 Espoo, Finland

**Keywords:** polymers, recycling, waste plastic, upcycle, circular economy, PET, additive manufacturing, distributed recycling, distributed manufacturing, 3D printing

## Abstract

This study explores the potential to reach a circular economy for post-consumer Recycled Polyethylene Terephthalate (rPET) packaging and bottles by using it as a Distributed Recycling for Additive Manufacturing (DRAM) feedstock. Specifically, for the first time, rPET water bottle flake is processed using only an open source toolchain with Fused Particle Fabrication (FPF) or Fused Granular Fabrication (FGF) processing rather than first converting it to filament. In this study, first the impact of granulation, sifting, and heating (and their sequential combination) is quantified on the shape and size distribution of the rPET flakes. Then 3D printing tests were performed on the rPET flake with two different feed systems: an external feeder and feed tube augmented with a motorized auger screw, and an extruder-mounted hopper that enables direct 3D printing. Two Gigabot X machines were used, each with the different feed systems, and one without and the latter with extended part cooling. 3D print settings were optimized based on thermal characterization, and both systems were shown to 3D print rPET directly from shredded water bottles. Mechanical testing showed the importance of isolating rPET from moisture and that geometry was important for uniform extrusion. The mechanical strength of 3D-printed parts with FPF and inconsistent flow is lower than optimized fused filament, but adequate for a wide range of applications. Future work is needed to improve consistency and enable water bottles to be used as a widespread DRAM feedstock.

## 1. Introduction

The vast majority of plastics end up landfilled or contaminating the natural environment, as the global polymer recycling rate is an embarrassingly low 9% [1]. The problems of plastic recycling were recently highlighted when China imposed an import ban on waste plastic [2], which stalled global recycling efforts [3,4,5]. Without China, large-scale centralized plastic recycling has become uneconomic in many cases, and many municipalities have stopped recycling [6]. Part of the problem is that it is costly to separate the numerous types of plastic, and as consumers have no direct financial incentive to do it in conventional centralized recycling, increasingly sophisticated sorting technologies are proposed [7] to reach a circular economy [8,9,10].

Another approach to reach a circular economy for plastic is Distributed Recycling for Additive Manufacturing (DRAM) [11,12,13]. In the DRAM methodology, consumers have an economic incentive [11,13] to recycle because they can use their waste as feedstock for a wide range of consumer products that can be produced for a fraction of conventional costs of equivalent products [14,15,16,17]. DRAM is a new technology that has the potential to radically impact global value chains [18]. Early DRAM work was centered on open source waste plastic extruders known as recyclebots, which upcycled post-consumer plastic waste into 3D printing filament [19,20]. In addition to reducing 3D printing costs by several orders of magnitude, it decreased embodied energy of 3D printing filament by 90% [21,22,23]. The open source 3D printing community, having evolved from the Self-Replicating Rapid Prototyper (RepRap) model [24,25,26], have embraced open source methods to recycle 3D printing waste [27] particularly for the two most popular fused filament materials: Polylactic Acid (PLA) [28,29,30,31] and Acrylonitrile Butadiene Styrene (ABS) [11,32,33,34,35]. More common thermoplastics were more challenging but have been successfully converted to filament including High-Density Polyethylene (HDPE) [19,36,37], Polypropylene (PP) and Polystyrene (PS) [37], Thermoplastic Polyurethane (TPU) [38], Linear Low-Density Polyethylene (LLDPE) and Low-Density Polyethylene (LDPE) [39], and Polycarbonate (PC) [40]. However, each melt and extrude cycle of a recyclebot impairs the mechanical properties of PLA [29], HDPE [41], and even of Polyethylene Terephthalate (PET) [42]. This limits the recycling cycles to approximately five [29] before reinforcement or blending with virgin materials becomes necessary. Polymer composites using carbon-reinforced plastic [43], fiber-filled composites [44,45], and various types of waste wood [46,47] have been used in recyclebot systems, and more complex DRAM systems can use 3D-printed PC as molds for intrusion molding [40] for windshield wiper composites [48] as well as Acrylonitrile Styrene Acrylate (ASA) and stamp sand waste composites [49]. Zander et al. [50] has studied PET, PP, and PS blends with Styrene Ethylene Butylene Styrene (SEBS) and maleic anhydride compatibilizers that were able to increase tensile strength from 19 to 23 MPa, although pure recycled PET had the highest tensile strength of 35 MPa.

This is part of the reason that the holy grail of DRAM has been PET. Although PET is only the 6th most commonly produced plastic, it is one of the most easily identifiable polymer waste streams for consumers [1] and is already widely recycled through centralized processes [51]. Although, the majority of centralized recycling is downcycling [52], it is, as noted earlier, not nearly at the rate to drive a circular economy. PET is an excellent water and moisture barrier, so it is used extensively in the packaging industry for consumable packaging of water, soft drinks, and foods [53]. PET use is expected to maintain a growth rate of 4.5%/year [54]. PET water bottles are easy to envision recycling at home as they are already clean and they are available in such large quantities globally as more than a million plastic bottles are produced every minute [55]. A few companies sell PET filament for 3D printing including Verbatim, MadeSolid, and Ultrafuse, and a few others sell recycled PET filament including Refil and B-PET. Although recycled PET and PETG filament is available commercially [56,57], some companies have stopped production [58,59]. PET is less popular than PLA, ABS, and PETG (glycol-modified version of PET) because the 3D printing process is more challenging as PET has shrinkage and warpage issues from high fusion temperature and lack of control of crystallinity, water absorption (leading to molecular weight reduction), and weak interfacial welding between layers [60]. In the past, PET industrial waste has been shown to successfully 3D print with Fused Particle Fabrication (FPF) or Fused Granular Fabrication (FGF) 3D printers that fabricate products directly from chips [61].

In centralized recycling, contamination and moisture are the major causes of deterioration of both the physical and chemical properties of PET [51]. In fact, Awaja and Patel state that to make a food-grade PET, the recycled PET must be hydrolyzed, purified, and re-polymerized [51]. Packaging-grade PET has been thought to require an increase in the viscosity or decrease in the melt flow index for effective use in material extrusion AM. One way to approach this is to use pyromellitic dianhydride chain extenders to increase the melt flow index of rPET via reactive extrusion [62]. There have been sporadic claims of PET recycling in the maker community, but they have failed to gain traction the way PLA or ABS recycling has caught on—primarily due to challenges in reproducibility. Part of these challenges also stem from the differences in the water bottles themselves, which are under constant flux. For example, since 2000, the average weight of a 16.9 ounce PET plastic bottle has declined by nearly half to 9.89 g, saving billions of pounds of PET resin [63]. The largest problem, however, has been identified as PET undergoes hydrolytic degradation during melt processing, resulting in reduced molecular weights and, if the feedstock is too wet, even total disintegration of the polymer. Fortunately, there has been progress, as Tech4Trade and other partners developed a complex custom recyclebot (the Thunderhead) specifically for PET recycling [64,65]. This system not only has a complex number and sophisticated heating zones, it also has a heated hopper to ensure that the feedstock flakes are always dry. The first systematic study of PET-based DRAM was conducted by Zander et al. in 2018 [60]. They found that the chemistry for different PET feedstocks was identical, and their rheological results showed that drying of the PET led to an increase in the viscosity [60]. Moreover, by altering the processing parameters, they were able to control crystallinity (24.9% for no active cooling down to 12.2% for the water-cooled filament) [60]. Finally, Zander et al.’s results of a PET tensile strength of 35.1 ± 8 MPa was found to be extremely promising as a material for DRAM [60].

To build on these promising results, this study explores the potential of PET packaging as a DRAM feedstock further by using only an open source toolchain with FPF/FGF processing. Earlier work showed that rPET pellets were processible [61], and here the more challenging flakes from rPET water bottles are investigated as a direct feedstock for FPF/FGF processing, without intermediary processing steps to convert the flake to pellets or filament, and without adding other materials to create composites. In this study, first the impact of granulation, sifting, and heating is quantified on the shape and size distribution of the rPET flakes. Then a feeding study was performed to determine whether they could be 3D printed through a feed tube connected to an externally mounted hopper, or whether the flake needed to be direct 3D printed with a gravity-fed hopper mounted to the print head. Two Gigabot X machines were used: one with extended part cooling and one without. 3D print settings were optimized based on DSC testing for the latter, and mechanical testing was performed on 3D-printed tensile bars. Both types of Gigabot X printers were used to fabricate example products from rPET pellets to show potential use cases for rPET 3D prints. The results are presented and discussed in the context of future work to make water bottles a DRAM feedstock.

## 2. Materials and Methods 

### 2.1. Materials 

**Figure 1:**
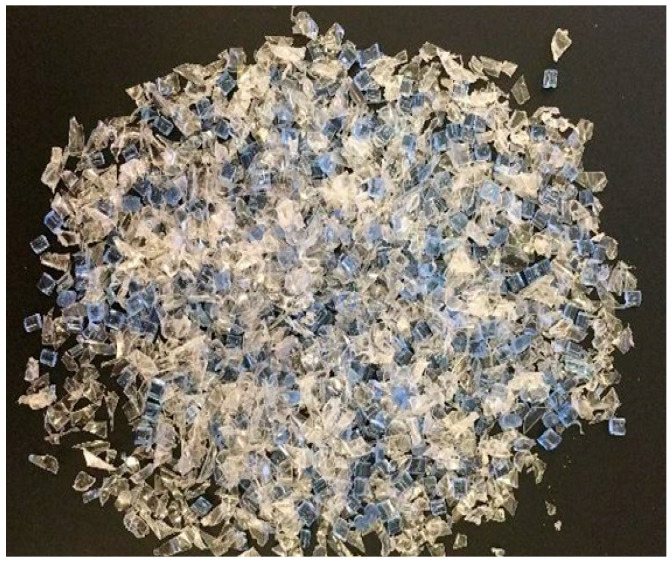
A 50:50 mix of Ultrafuse (blue pellets) and shredded water bottle (clear) to show relative, size, shape, and texture.

Two recycled PET (rPET) materials were tested (see Figure 1). First, Ultrafuse PET pellets, which have previously been shown to be conducive to FPF/FGF processing with a Gigabot X prototype [61] with two hot zones were evaluated. This commercial recycled rPET was shown to have ideal temperature settings of 220 and 230 °C for zone 1 and 2 respectfully. The print bed was set at 100 °C and printing speeds from 5 to 30 mm/s were all shown to be adequate [61]. The second material was granulated water bottles. Water bottles were collected in Houston, Texas, and consisted primarily of the brands Hill Country Fare (0.11 mm thickness), Great Value (0.092 mm), Ozarka (0.09 mm), and Texas Music Water (0.09 mm). Thickness measurements were taken using calipers at the top domed section of the bottles, where no seams were present. To convert PET water bottles into 3D-printable regrind material, the labels, caps, and adhesives were removed before granulating the bottles in a SHINI USA open rotor scissor cut granulator [66]. The granulator produced regrind small enough to pass through its grate, which has 5.84 mm diameter holes. After granulation, the regrind was dried in a food dehydrator for 24 h at 38 °C. This temperature was chosen to ensure that the rPET was not degraded by the drying process. In Figure 1, the pellets (blue) are not only more uniform but also bulkier than the granulated water bottle rPET (clear granulate).

### 2.2. Granulate Particle Analysis

To compare the different PET sources, the granulate were characterized using FIJI ImageJ software [67]. The size characteristics of the particles for each starting material were quantified using digital imaging, and the open source Fiji/ImageJ Circularity, *c*, was defined as [68]:

(1) $$c=4\pi \times \frac{A}{P^{2}}$$

where *A* is area in mm and *p* is perimeter in mm. Thus, a circularity value of 1.0 indicates a perfect circle; whereas as the values approach 0, it indicates an increasingly elongated polygon. Then to compare the different processing methods for the PET water bottle granulate, the cross-sectional areas of granulate particles were plotted as normal distributions and compared. 

### 2.3. rPET Thermal Materials Characterization

The thermal properties of rPET flake were first characterized with Differential Scanning Calorimetry (DSC) in order to have a starting point for the 3D printing process parameter optimization. Untreated rPET flake samples from post-consumer water bottles that were scanned for the DSC were then used as described in Section 2.4 and Section 2.5. The rPET flakes were tested three times using the Netzsch DSC 404 furnace under pure argon flow of 50 mL/min and a heating rate of 10 °C/min. Background scans were performed on an empty aluminum crucible for each sample which generated calibration curves used to normalize the scan. The sample masses were measured with a precision of ±0.01 mg on a Sartorius scale and then entered into the Netzsch software. During the tests, each PET sample was placed into the aluminum crucible pan alongside an empty reference pan, and then the furnace chamber was purged and backfilled with argon to ensure no oxygen was present. Following this, the instrument heated the pans starting at 30.0 °C ± 7.5 °C. Heating at a constant rate of 10 °C per minute, the crucibles were brought to a temperature of 300 °C and then cooled back to room temperature. 

### 2.4. FPF/FGF 3D Printing

**Figure 2:**
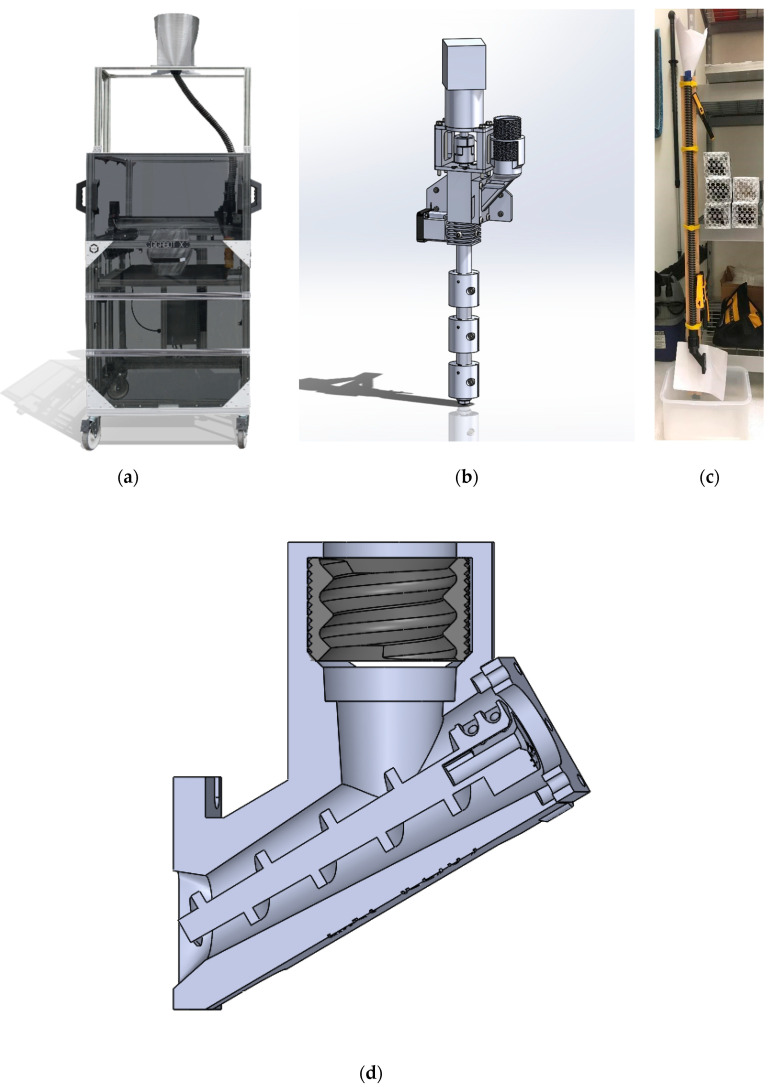
(**a**) Gigabot X design with feed tube, (**b**) close up of 3-heat zone extruder, (**c**) feed test apparatus and (**d**) cross-section view of the Crammer feed throat with the auger screw inside.

Two approaches were taken to 3D print with flaked water bottles directly without first converting the rPET into filament. In the first approach a feed tube arrangement (Figure 2a) was used with a 3-heat-zone Gigabot X (re:3D, Houston, TX, USA) (Figure 2b). The Gigabot X is a direct pellet material extrusion-based 3D printer, with the nozzle arranged vertically as in Figure 2b. A compression screw and three hot zones enable a relatively constant flow of recycled material through the print nozzle. The turning of the compression screw acts the same as the main feed motor for a fused filament type RepRap machine. To assess the ability for a material to 3D print on Gigabot X design (Figure 2a), consistent flow through the 3D printer’s feeding system was evaluated. Therefore, all samples of processed granulate were subjected to feed tests to identify which samples flowed through both the feed tube and feed tube adapter. A testing device was built with these components (Figure 2c). To perform the feed test on a material sample, the following steps were used:

1. Blocked bottom end of the feed tube.
2. Loaded the feed tube from the top with test material until it is full.
3. Unblocked the bottom of the feed tube to allow material to flow through via gravity.
4. Recorded whether all the material flowed through or became stuck inside the tube.
5. Repeated with the feed tube adapter attached at bottom of the feed tube to measure material flow through both the tube and adapter by massing the material as function of time.

To determine the 3D printing temperatures and test the extrusion rate of PET flake, the extruder was first flushed with Ultrafuse recycled PET (rPET) commercial pellets [69]. The PET water bottle flake was then fed directly into the feed throat to eliminate any effect of inconsistent granulate flow through the feed tube (Figure 2c). Initial extruder temperatures were set to 250 °C for the bottom heating zone closest to the nozzle, 240 °C for the middle, and 180 °C for the top. The bottom zone temperature was set by incrementing up at 5 °C intervals until the granulate at the nozzle began to flow, and the top temperature was set low enough for the granulate at the top of the pellet screw to remain unmelted and provide pressure to extrude the melted plastic lower in the screw.

To flush the Ultrafuse rPET pellets out of the extruder and transition to extruding the rPET granulate, the extruder motor was rotated in increments of 200 mm, at 600 steps/mm and at a speed of 3 mm/s. The use of millimeters in both the 3D printer firmware and in Simplify3D is designed for filament 3D printing, and describes the length of filament pulled by the motor and extruded. When open source firmware and slicing are developed specifically for direct-drive FPF, these values can be converted to rotations per minute using the steps per revolution for the motor to be consistent with what is occurring physically. When flushing from rPET pellets to PET water bottle granulate, the granulate did not extrude reliably. When flushing from water bottle granulate to rPET pellets, consistent extrusion was achieved. This indicated feeding issues due to the physical particle characteristics.

Therefore, additional processing methods were explored to decrease particle size and increase particle sphericity. This is because it is well known that spherical particles flow most easily [70], and although the impact of size on flow of particles is complex, in this system the smaller the particle size would have a lower probability of becoming jammed and restricting flow in the feeding tube. The processing methods explored to improve feeding and printability include:

1. Granulating Twice: Feeding granulated water bottles back into the SHINI granulator [66].
2. Sifting: Sifting through a 3D-printed sifter [71] with holes 5 mm in diameter and 2 mm deep. Sifting removes 40% of the granulate by weight, producing a 60% yield.
3. Heating:
  a. Heating in a food dehydrator at 65.5 °C for 24 h.
  b. Heating in an Analog Air Forced Analog Lab Oven (Quincy Lab) at 100 °C for 1 h.
4. Sequential sifting (2) and heating (3b): Sifted through the 5 mm hole sifter, then heated in the oven at 100 °C for one hour.

Additional tests were conducted to further quantify factors affecting particle shape when heated. Fiji/ImageJ measures a curled particle as having a smaller cross-sectional area than if the same particle were flattened. To better measure particle area changes without the factor of curling, flat 25.4 × 25.4 mm square samples (6.45 cm^2^) were cut from the top portion of water bottles and submitted to various heating tests. To evaluate the diversity of plastic PET previously reported, five different brands of water bottles were assessed: Baraka, Hill Country Fair, Great Value, Ozarka, and Texas Music Water. Baraka bottles were sourced from a U.S. Air Force Forward Operating Base, and the others were sourced in Houston. Samples of each brand were heated at 100 °C for 1 h. After the heat cycle, dimensions were measured while the samples squares were flat. Tests for time and temperature dependence on plastic sample dimensions were also performed on water bottle brands Baraka (average thickness 0.25 mm) and Ozarka (average thickness 0.2 mm):

1. Time dependence: heating at 100 °C for varying lengths of time.
2. Temperature dependence: heating for 5 min at temperatures ranging from 60 to 100 °C.

Finally, to improve feeding of water bottle rPET flake and other nonuniform regrind into the Gigabot X extruder, a Crammer (Figure 2d) was developed. The re:3D Crammer is a motorized auger screw that mounts onto the extruder and physically pushes the rPET flake from a feed tube and into the extruder. The Crammer’s motor is synced with the pellet extruder motor via the duplicate nozzle, or ditto printing, feature of the open source Marlin firmware. This allows the Crammer to convey flake whenever the main extruder extrudes, and to scale the rate of material conveyance with the extrusion rate of the main extruder. The Crammer’s components are all 3D printed from polycarbonate and were made available as open source designs [71].

Typical auger screws convey material with a screw that fits snugly in a barrel, preventing any material from passing between the screw flighting. This style of screw was tested with TPU pellets and successfully conveyed them. However, water bottle flake would get caught between the screw and the internal walls of the feed throat, stalling the Crammer motor and preventing conveyance. Since higher tolerance between the parts could not be achieved with 3D printing, an alternative screw was designed that left space between the screw threads and the feed throat internal walls, allowing flake to pass between the screw flighting without getting stuck (Figure 2d). This design was able to convey the water bottle flake without the flake getting trapped between any of the components.

**Figure 3:**
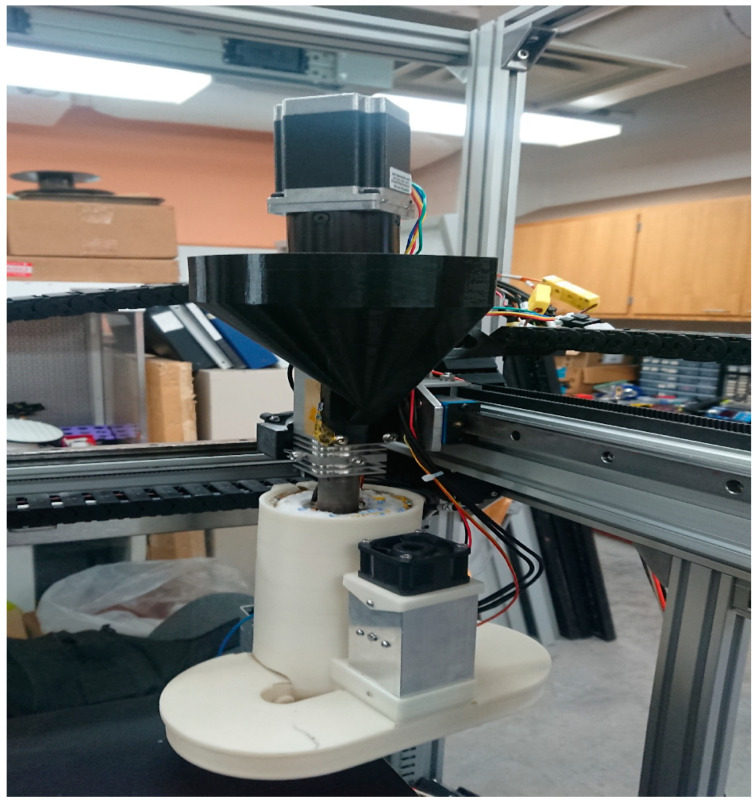
Gigabot X with 3D-printed direct feed hopper (black) and 3D-printed cooling shroud (white) with the source code available [71].

A second feeding system approach was also tested on the granulated water bottles. A Gigabot X was outfitted with an extruder-mounted hopper, a 1.75 mm printer nozzle diameter, and a 3D-printed part cooling arrangement shown in Figure 3. The design files for the cooling setup can be found on the Open Science Framework [71]. 

### 2.5. Printing Settings Optimization

Using the extruder-mounted hopper and 3D-printed cooling setup in Figure 3, 3D print optimization was performed on two geometries:

1. Cylinder: 20 mm diameter, 40 mm length, slicer generated mass of 17.3 g.
2. Cuboid: 50 mm length, 50 mm width, 5 mm height, slicer generated mass of 17.3 g.

Following similar protocols to those established by Woern et al. [61], optimization was performed in the 180 to 260 °C region, with the heater region closer to the nozzle always having an equal or higher temperature. The minimum temperature at the top of the feeder was chosen based on the motor skipping. This indicates that the torque required to turn the extruder screw is higher than the extruder motor’s torque output, which usually means material is unmelted or highly viscous. A temperature where there was no motor skipping was chosen (210 °C). The maximum temperature close to the nozzle was chosen based on the blob-like appearance of the 3D print due to melting of the material (240 °C). Specimens were 3D printed at various temperature combinations in the selected “printable” region, and the chosen geometries were optimized based on visual quality and mass of the specimen. Three print speeds were tested: 10, 30 and 50 mm/s.

### 2.6. Mechanical Testing

Two sets of mechanical testing took place. First, on the feed system shown in Figure 2 with rPET water bottle flake. Before loading into the 3D printer, the water bottle flake was dehydrated at 100 °F for 24 h, then placed in a 180 °C oven for 5 min to improve feeding based on the results of the water bottle flake testing. With the Crammer, the water bottle flake could be extruded enough to produce ASTM D638 Type I tensile bars with a 0.8 mm nozzle and a 0.6033 mm layer height. Tensile bars were pulled on an Admet eXpert 2600 with a tension test with extensometer setup using the ASTM D638 testing standard. Five (5) specimens were tested in each sample. Specimens were massed on a digital scale.

Second, tensile testing was completed on the Ultrafuse rPET pellets (not PET flakes) using the ASTM D638 Type 1 standard tensile bars on the second set up in Figure 3. The nozzle size was 1.75 mm. layer height 1 mm. The bars were 3D printed at ideal print settings that were found during optimization (see Appendix A) of the cuboid at 100% infill. The infill grid pattern was set to 45 degrees with respect to the long axis of the tensile bars. Five (5) specimens were tested in each sample. The specimens were then pulled until failure using a 10 kN load cell on an Instron 4210 Testing machine and the speed of testing was 5 mm/min. The strain data were captured using the crosshead of the Instron 4210. All mechanical testing was at room temperature (23 °C).

## 3. Results and Discussion

### 3.1. Particle Size Analysis of Granulate and Feeding

**Figure 4:**
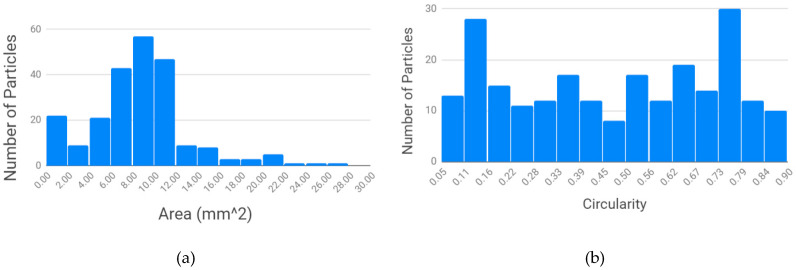
Ultrafuse rPET: particle size distribution (**a**) and particle circularity as a function of area (**b**).

For the Ultrafuse rPET pellets shown in Figure 4, the average area in Figure 4a was 8.73 mm^2^ and the median area was 8.57 mm^2^, with a standard deviation of 4.59. The average circularity for the Ultrafuse rPET in Figure 4b was 0.47 and the median was 0.50, with a standard deviation of 0.25.

**Figure 5:**
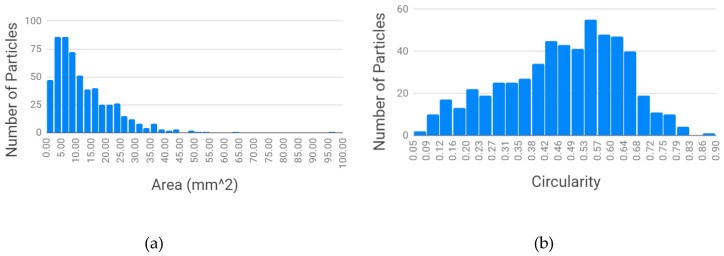
Houston-sourced PET water bottle flake: particle size distribution (**a**) and particle circularity (**b**).

As shown in Figure 5, the average area in Figure 5a was 12.56 mm^2^ and the median area was 9.27 mm^2^, with a standard deviation of 10.43. The average circularity for the unscreened water bottle rPET in Figure 5b was 0.47, the median was 0.49, with a standard deviation of 0.17.

**Figure 6:**
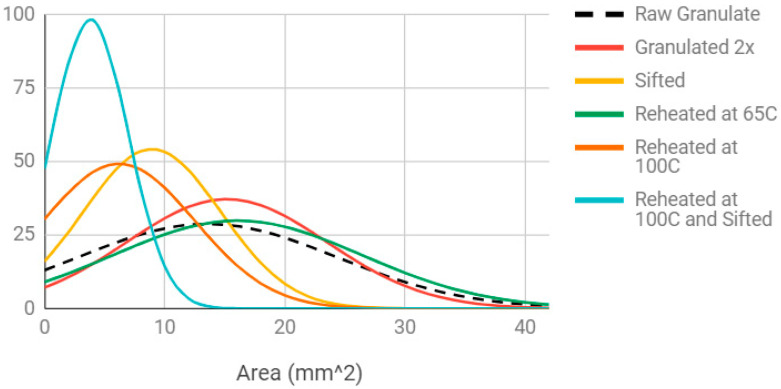
Effect of different processing methods on the normal distribution curves for PET water bottle granulate particle area.

By comparing the results of the two materials in Figure 4 and Figure 5, the particle area of the Ultrafuse pellets is substantially smaller than the recycled water bottle granulate, as is the standard deviation. The circularity of the two materials is equivalent. A clear approach to improving the 3D printability of the recycled water bottle PET is simply to reduce its size. The impact of the four approaches to reduce the size of the rPET water bottle granulate is shown in Figure 6. 

As shown in Figure 6, the ImageJ particle analysis revealed the following conclusions for the different processing methods:

1. Granulating twice: Passing the water bottle granulate through the SHINI granulator twice does not decrease particle size (Figure 6). In fact, it shifts the particle size distribution to the right, toward larger particles. This indicates a loss of smaller particles (<2 mm^2^ in area) in the granulator. Not only does this processing step take more time and energy, it is ineffective.
2. Sifting: Sifting successfully reduces the average particle area from 12.56 to 9.14 mm^2^ and shifts the particle size distribution curve to the left (Figure 6). This is a promising method for obtaining a 3D-printable granulate from rPET water bottles, but results in additional waste plastic.
3. Heating: Heating at 65 °C does not reduce particle area and instead slightly shifts the normal distribution curve to the right (Figure 6). This may indicate a loss of small particles in the dehydrator during the heating process, since the smallest particles can fall through the dehydrator’s screen holes. However, heating at 100 °C in the oven does reduce particle area (Figure 6), presumably because the flat plastic particles curl and contract in area while also increasing in thickness. The sample heated at 100 °C also contained some particles that underwent a color change from clear to opaque white. The shape and color changes indicate crystallization of the amorphous PET water bottle plastic. Crystallization begins at the glass transition temperature (Tg), which for PET is in the range of 153–178 °F (67–81 °C) [72]. This explains why the shape and color changes were present in the PET heated at 100 °C (above Tg) and not in the PET heated at 65 °C (below Tg).
4. Combined sifting and 100 °C heating: Finally, the combined approach was shown to further tighten the particle size distribution and shift it towards smaller particles as shown in Figure 6.

**Figure 7:**
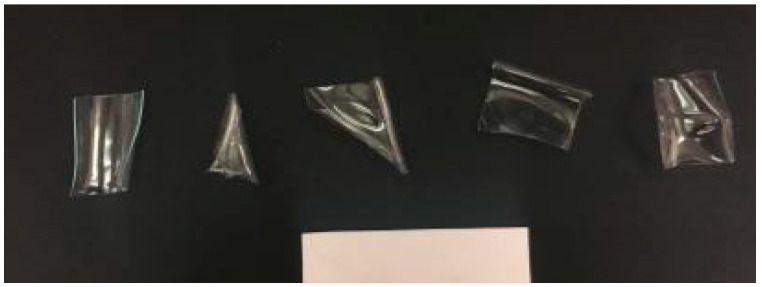
Water bottle squares after 1 h at 100 °C (scale shown with 50.8 mm white bar).

To investigate the impact of different water bottle sources on rPET properties when heated, squares cut from various brands of water bottles were heated at 100 °C for 1 h (Figure 7). After heating, their dimensions were measured while the squares were flat, and the percent change was found (Table 1).

PET samples heated to 100 °C underwent significant contractions in length and width across all water bottle brands. The sample squares contracted different amounts in each dimension, with an average percent change of −17.4% in one dimension and −5% in the other (Table 1). The difference between the two dimensions may be caused by the water bottle manufacturing process, but more investigation is needed to confirm. This indicates that heating above the glass transition temperature shows promising results in improving particle shape.

**Figure 8:**
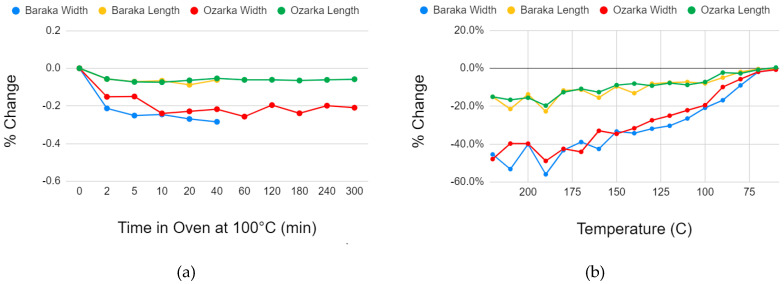
Effect of heating time on dimensions (width and length) of 25.4 mm squares of PET water bottle plastic heated at 100 °C (**a**) and the effect of temperature on dimensions (width and length) of 25.4 mm squares of PET water bottle plastic heated for 5 min (**b**).

Heating tests for time dependency on PET sample dimension show that area reduction occurs within the first five minutes, and additional heating time does not provide additional particle shape benefits (Figure 8a). By contrast, in Figure 8b, area reduces as temperature increases, beginning at the glass transition temperature (Tg) of PET. Area changes were similar across water bottle brands Baraka (avg thickness 0.25 mm) and Ozarka (average thickness 0.2 mm). These experiments also confirmed that the percent change in width was consistently double than that in length.

**Figure 9:**
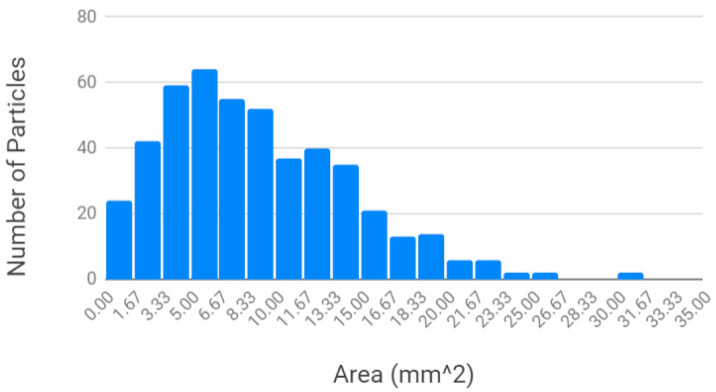
Particle size distribution of rPET water bottle flake sifted, then heated at 190 °C for 5 min.

Based on conclusions from the temperature and time dependence tests, a sample of rPET water bottle flake was sifted, then heated at 190 °C for 5 min to obtain a sample with the smallest cross-sectional particle area (Figure 9 as compared to results in Figure 6).

Although the combined sifting and heating at 190 °C had the best chance of providing a functional material for the Gigabot X, the feeding tests showed that it was still incompatible. Although the Ultrafuse rPET pellets were easily fed through feed throat and 25.4 mm tubing, the processed samples of PET water bottle flake did not consistently feed through the system. This severely impacted 3D printability via the feed tube, and led to the development of the Crammer, or motorized auger screw (Figure 2), to physically push the water bottle flake from the feed tube and into the extruder. This system enabled post-consumer water bottle-sourced rPET flake to be directly 3D printed.

### 3.2. Thermal Analysis

**Figure 10:**
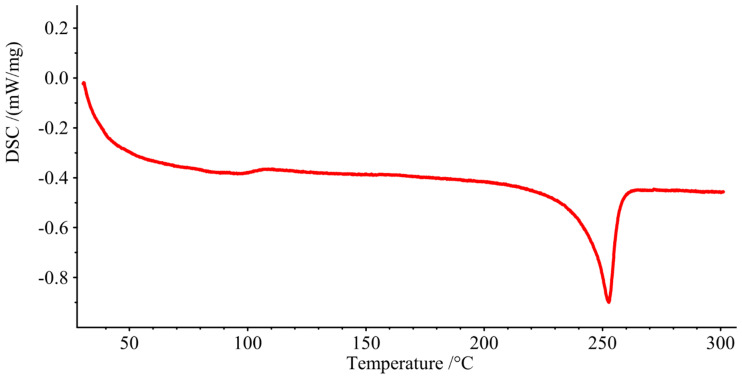
DSC curve of shredded water bottle.

Figure 10 show the DSC curve for a sample of PET water bottle mW/mg as a function of temperature (°C). The positive y axis indicates exothermic reactions, while downward designates endothermic reactions. The PET water bottle samples have endothermic peaks after approximately 250 °C, which indicates a melting peak, showing that the PET flakes have a melting temperature of approximately 250 °C, which is what is expected of PET resin [73].

### 3.3. 3D Printing

#### 3.3.1. Optimization Results

The optimum temperature settings for 3D printing were found for the approach shown in Figure 3 in Table 2 (details of all runs available in Appendix A). It should be noted, however, that any specimen 3D printed in the given temperature range was sufficiently good in visual quality and mass of the 3D print.

Specimens 3D printed at high speeds were consistently underextruded for various temperatures. Hence, a low speed of 10 mm/s was chosen as the ideal print speed.

#### 3.3.2. Mechanical Testing

**Figure 11:**
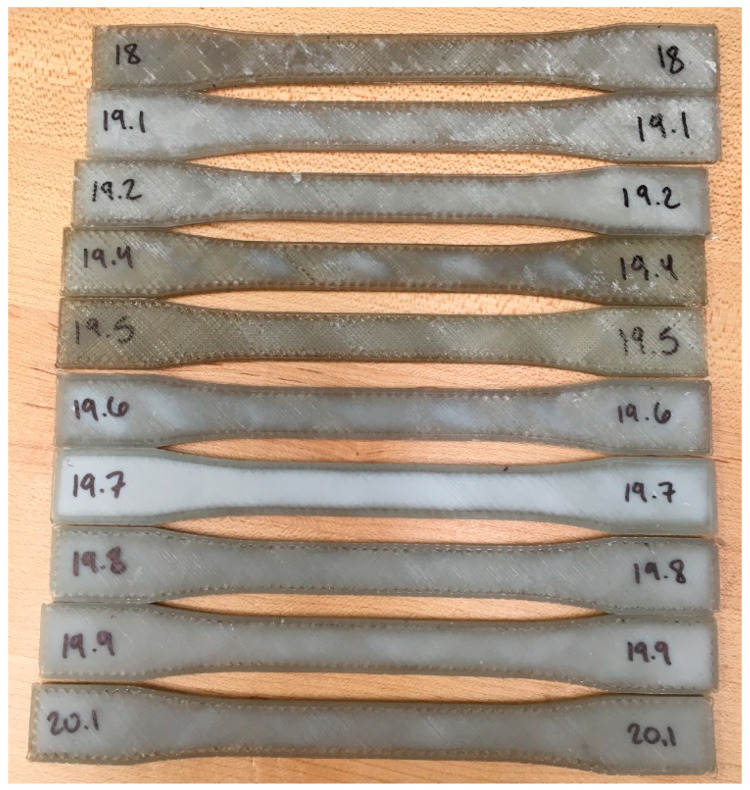
Tensile bars 3D printed with rPET water bottle flake on the adapted Gigabot X and Crammer shown in Figure 2.

Water bottle rPET flake was successfully 3D printed into tensile specimens (Figure 11) with an adapted Gigabot X and Crammer shown in Figure 2. The average Ultimate Tensile Strength (UTS) of these direct 3D-printed materials was found to be 20.35 MPa, with a standard deviation of 0.187 MPa. 

**Figure 12:**
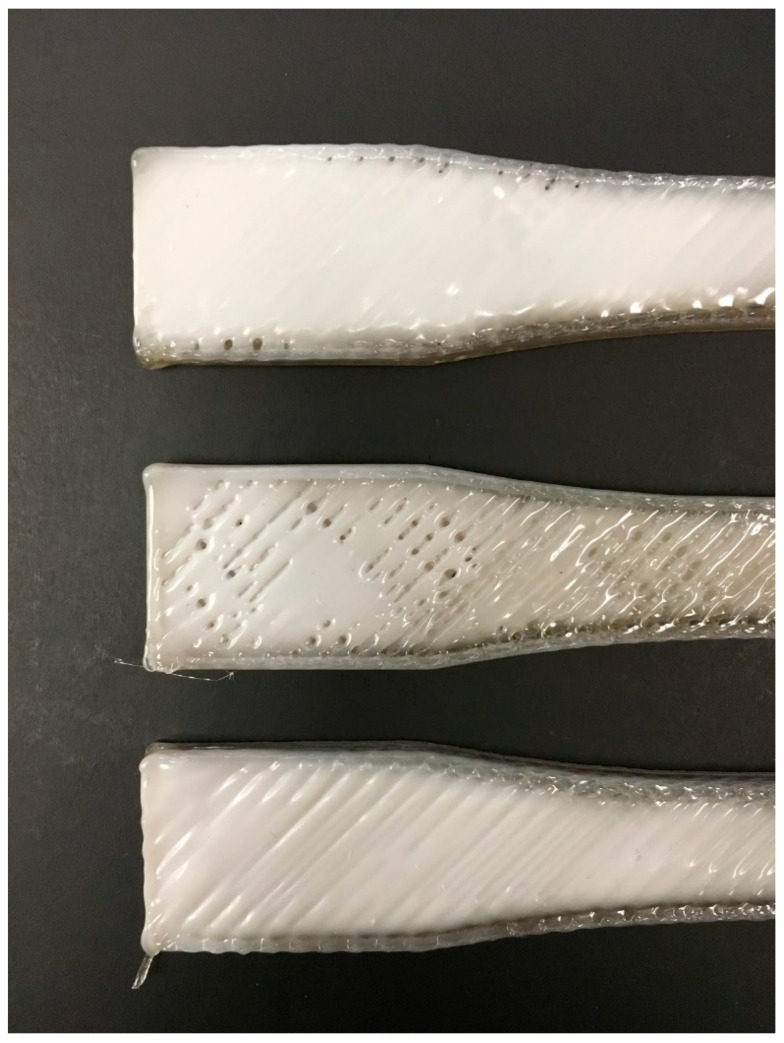
Close up of rPET water bottle tensile bars 3D printed with the same print settings. Inconsistent extrusion caused both under extrusion (top, middle) and over extrusion (bottom), with under extruded sections resulting in macro voids that decreased the UTS of the tensile bar.

The variance in the UTS values and the resulting standard deviation may be a result of macro voids in the rPET tensile bars. These voids are caused by inconsistency in extrusion throughout a single 3D print (Figure 12). This caused a range in mass of the samples. The samples had an average mass of 10.4 g, with a standard deviation of 0.76 g. Optimization of the Crammer and further research into improving rPET flake extrusion may resolve the macro voids and improve the UTS of rPET flake to be more comparable with virgin PET, or rPET fabricated first into filament.

**Figure 13:**
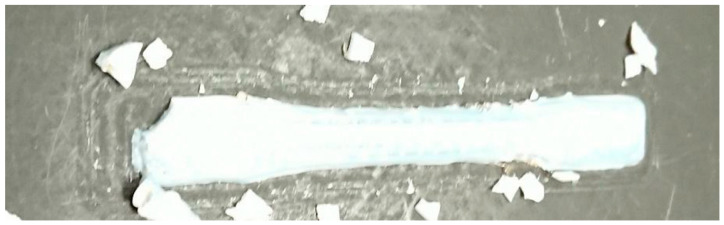
Tensile bar 3D printed in rPET from water bottle flake fractured on bed removal if 3D printed in humid open air.

In addition, using the second feed system (shown in Figure 3) was attempted. Although rPET from flake was found to be 3D printable via direct hopper, the lack of reproducibility and extreme brittleness resulted in a nonviable 3D print for tensile testing, since it could not be removed intact from the build surface, as shown in Figure 13.

Using the second feed system approach, Ultrafuse rPET pellets were tested with and without a cooling fan shown in Figure 3. The average tensile strength of the rPET pellets 3D printed with a cooling fan was 12.93 MPa, with a standard deviation of 4.72 MPa, while the average tensile strength of the sample 3D printed without using a cooling fan was 25.32 MPa, with a standard deviation of 5.82 MPa. The use of a cooling fan clearly reduced the tensile strength of the specimen although it provided more accurate 3D printing of small features as the forced cooling locked the molten plastic into the 3D form and reduced oozing. It is also observed that the average mass of the sample 3D printed using a cooling fan was 9.5 g, while it was 9.2 g without using a fan. This mass discrepancy is most likely attributed to the variance in feeding and thus porosity of the direct feed process. The higher porosity in the cooling case reduced the UTS similar to the observed effect from inconsistent feeding observed for the first feed system approach and rPET flake. 

To provide a comparison for the tensile bars 3D printed from water bottle flake, the Ultrafuse rPET pellets were also 3D printed on the first feed system setup (Figure 2) and the results were found to be more uniform. This resulted in a UTS of 29.62 MPa and standard deviation of 4.43 MPa. The same nozzle (0.8 mm) layer height and layer settings were used for the rPET flake. The highest value observed is within the range previously reported for PET water bottles in a scientifically controlled environment using preformulated filament that enables better control of the material extrusion [60]. PET has a bulk tensile strength of 47 to 90 MPa [74], whereas Zander et al. have shown that the strength of 3D-printed PET ranged from 27 to 45 MPa [60]. Woern et al. have shown rPET in a Gigabot X prototype produced an average tensile strength was 40 MPa [61]. Overall the UTS observed in this study for direct 3D printing water bottle flake was approximately equivalent to half that observed for Zander et al., which used a two-step process that first extruded filament and then 3D printed it. Zander et al. also showed that improved properties were possible forming waste composites [60], which is a clear potential to be successful for this direct shredded waste FPF 3D printing approach shown here. In addition, mixing rPET from flake and industrial rPET pellets or mixing with polypropylene to form blends [75] may lead to improved mechanical strength for FPF and can be investigated in the future.

Further study must be performed to understand the reason for this observed brittleness of the rPET from water bottle flakes as well as the variable strength of rPET pellets observed here although a few hypotheses can be made. First, the propensity of PET to break down in the presence of water and heat is well known. Although the PET was dried before entering the open hopper, the humidity in the room would have enabled access to water. Depending on the print order of the sample, the rPET plastic could have been held at elevated temperatures within the extruder of the Gigabot X, partially breaking it down. This may indicate why previous results with a shorter two stage Gigabot X hot end (and thus a shorter high temperature residence time) resulted in higher tensile strengths for rPET [61]. This brings us to the second explanation—that the results indicate that there is wide variety in the quality of PET water bottle plastic and this plastic could have been of the less mechanically or chemically stable variety. The most perplexing result is that the strengths with cooling were approximately half those without cooling. This is not expected as a faster cooling rate would typically result in higher strength. The average mass with cooling was also larger by approximately 3%, which would have also indicated that it would be stronger. The nature of material extrusion-based 3D printing may also help to explain this result. The observed macro porosity is a strong indicator that this was the primary explanation of both results. If the plastic in both cases was approximately the same (or even slightly higher for the cooling fan case), the rapid cooling could create more interline spacing (triangular shaped air gaps). As previously observed in FFF printing [76], these gaps would be expected to reduce strength even if the 3D prints appeared solid. In addition, because the two cases that were tested for tensile strength were no cooling and modest cooling, slow print speeds were necessary (10 mm/s), which would be expected to increase any breakdown in the material in the 3D printer. 

#### 3.3.3. Example Print

**Figure 14:**
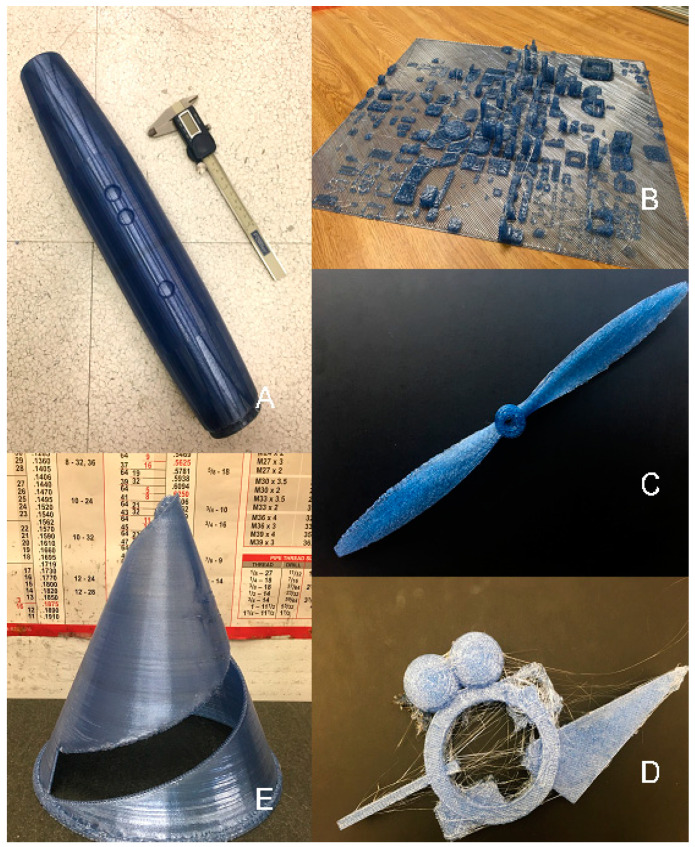
Example Gigabot X test 3D prints: (**A**) Air Force training aid, (**B**) topographical map KMZ, (**C**) propeller, (**D**) planning tool, and (**E**) spinner.

The three-heating stage Gigabot X was able to fabricate several example 3D prints with Ultrafuse rPET pellets. Despite the rPET being substantially weaker than injection molded PET, the values of 19.5 and 25 MPa are close to those observed for commercial FDM of ABS plastic as well as FFF ABS 3D printed under realistic conditions [77]. This makes the rPET even FPF 3D printed directly from flake more than adequate for a number of applications. Several examples are shown for military tools and training aids in Figure 14: (A) Air Force training aid: successfully 3D printed with a 0.8 mm nozzle and no support; (B) KMZ topographical map: 3D printed first with a 1.75 mm nozzle, then a 0.8 mm nozzle to improve resolution; (C) propeller: 3D printed with a 0.8 mm nozzle with support (surfaces contacting the support can be improved with higher resolution and dual extrusion); (D) planning tool: the combination of support and high-detail parts could not be achieved with the resolution from either the 1.75 or 0.8 mm nozzles; (E) jet engine jig to paint the white line on a spinner: successfully 3D printed with a 1.75 mm nozzle with vase mode. Overhanging edges can be further improved by a smaller nozzle and this would also solve the quality issues shown in Figure 14B.

**Figure 15:**
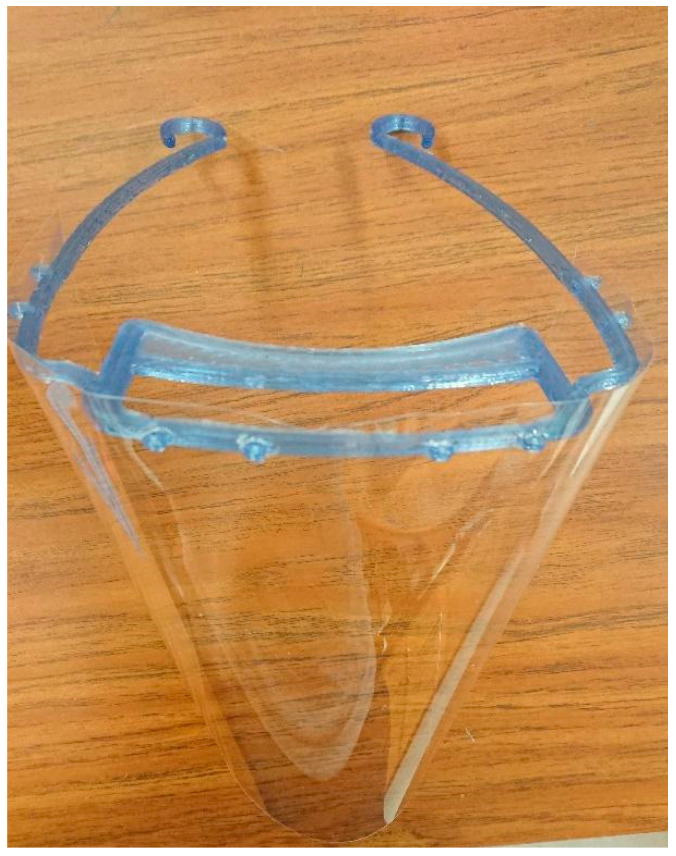
Recycled PET pellets used to 3D print a face shield.

To further demonstrate the feasibility of using rPET to 3D print a high-demand object [78,79,80,81], the Gigabot X was used to 3D print a face shield as shown in Figure 15.

## 4. Future Work

This study has uncovered several areas of future work. First, improved methods of granulating PET from water bottles as well as processing rPET flake into an FPF/FGF machine are needed. This is expected to help feeding issues observed in this study with the Gigabot X feeding tube system which, unlike the extruder-mounted hopper system, enables large-scale, long-term 3D printing, and already works with uniform feedstocks such as pellets. Future investigation into improving feeding issues for water bottle flake and similarly shaped recycled flake can include flake processing techniques, feed system part geometry to improve flow, and improvements to the motorized auger screw to physically pack particles into the extruder. Second, the results of this study showed that there is a large difference in the rPET with different waste streams, processing history, and physical form (flakes or pellets). This could be a function not only of the supplier and their feedstocks and additives, but could also be influenced by age of the waste, whether it was stored in direct sunlight, and the thermal history. This represents a substantial challenge to the optimization of the DRAM process. One approach to partially solving this challenge is to expand the ‘Consumer Bill of Rights’ to include material ingredient lists maintained in a freely accessible digital database for all consumer products [82]. In addition, these rPET materials should be subjected to detailed rheological analysis and the necessity of viscosity enhancing additives can be explored. This can be for the pure rPET materials as well as mixtures of pellets and shredded water bottles. This is a complex problem and a far more detailed study should be completed looking at rPET from many sources, locations in the world, and suppliers to provide optimal 3D printing parameters for direct extrusion 3D printers such as the Gigabot X. One of the first steps could be the development of an open source melt flow index (MFI) device that could be used to rapidly screen rPET materials at a low cost. This would partially overcome the challenge of not knowing the history of a rPET material (even if the full chemical makeup was supplied by the manufacturers). 

Going beyond more complete knowledge of the rPET material, the FPF 3D printing process can also be improved. Additional work is also needed to quantify the impact of nozzle height and layer height on the strength of rPET 3D prints as well. In order to overcome the slow printing speeds used in this study, a more powerful fan could be added to the system to enable rapid part cooling. This would be expected to allow for faster 3D printing (reducing residence time and reducing material breakdown). Detailed study measuring the crystallinity of the 3D-printed specimen is required to understand the reason for the difference in tensile strength between specimens 3D printed with and without a fan. To better understand the material breakdown, a careful study of residence time vs. strength could be completed for future work. In addition, as with PLA [29], ABS [83], and HDPE [84], the impact of the number of recycle loops should be investigated and compared to a filament-extruding approach for multiple cycles. This is important to have a closed-loop supply chain in the circular economy [12,85]. It is likely that the direct rPET FPF 3D printing demonstrated in this study would provide an advantage, as the number of melt/solidification loops would be reduced by ½. Finally, in order to ensure that the rPET remains dry, a heated hopper/feeding unit could be investigated and would be expected to improve results. This work should enable rPET from water bottles to be used as a reliable feedstock for DRAM.

## 5. Conclusions

Although far from optimized, the results of this study show the potential to reach a circular economy for post-consumer recycled rPET as a DRAM feedstock when used with Gigabot X FPF/FGF 3D printing. The results showed that extended feeding tubes were challenging with rPET flakes when processed by simple shredding, sifting, or heating (and the combination), but they could be 3D printed using a Crammer to improve feeding, and resolution could be improved with active cooling. Further, this study showed a wide disparity in the physical properties of rPET depending on source and particle shape (flake or pellet) and indicated a large area for future work both in material characterization as well as processing and machine design to make rPET from water bottles a common feedstock.

## Figures and Tables

**Table 1:** The 25.4 mm water bottle squares before and after a 1 h heat cycle at 100 °C.

	Post-Thermal Treatment			Percent Change	
Bottle Brand	Width (mm)	Length (mm)	Area (mm^2^)	Width	Length
Baraka	17.8	23.9	424.5	−30.0%	−6.0%
Hill Country Fare	22.1	23.1	510.8	−13.0%	−9.0%
Great Value	21.3	24.4	520.3	−16.0%	−4.0%
Ozarka	21.3	24.6	525.7	−16.0%	−3.0%
Texas Music Water	22.4	24.6	550.7	−12.0%	−3.0%

**Table 2:** Optimal 3D print settings for the three temperature zones of the Gigabot X for no-fan and small-fan cases for rPET flake (Figure 3).

Cooling Fan	Shape of Print	Temperature (°C)		
		Bottom	Middle	Top
No fan used	Cylinder	210	200	200
	Cuboid	230	230	220
Small fan used	Cylinder	220	220	210
	Cuboid	230	220	220

**Figure A1:**
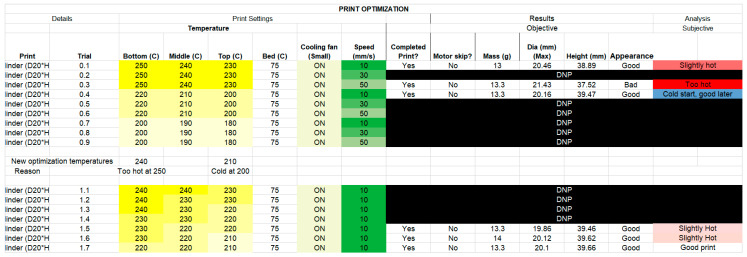
Print optimization with small-fan cylinder.

**Figure A2:**
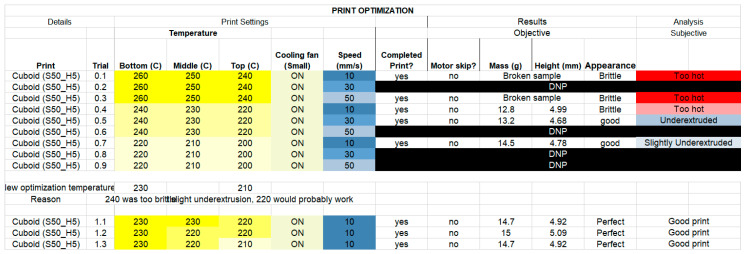
Print optimization with small-fan, large short cuboid.

**Figure A3:**
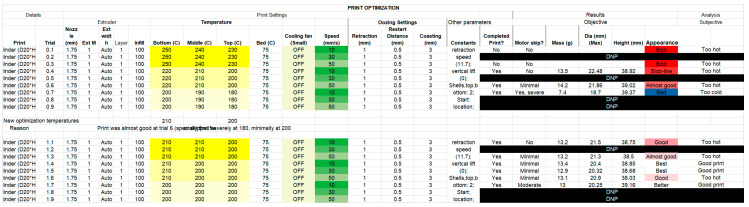
Print optimization with no-fan, small tall cylinder.

**Figure A4:**
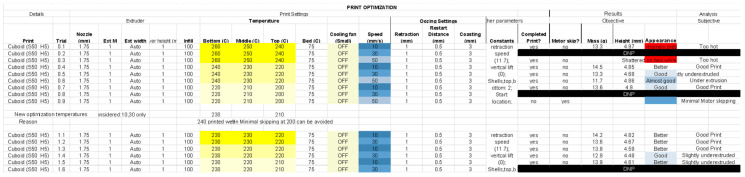
Print optimization with no-fan, large short cuboid.
